# Étude comparative de patients hospitalisés pour une infection SARS-CoV-2 au cours de deux vagues consécutives en Tunisie

**DOI:** 10.48327/mtsi.v2i3.2022.207

**Published:** 2022-08-18

**Authors:** Rim RACHDI, Souha HANNACHI, Sabrine ZRIBI, Oumaima AYED, Rim ABID, Zied MOATEMRI, Samira MHAMDI, Salsabil DABBOUSSI, Hédi GHARSALLAH, Walid SELLAMI, Walid SAMMOUD, Hakim MASSOUDI, Khaled LAMINE, Olfa DJEBBI, Rim HAMMAMI, Mohamed BEN MOUSSA, Ridha BELLAAJ, Riadh BATTIKH, Mohamed Radhouane RACHDI, Mustapha FERJANI

**Affiliations:** 1 Service d'hygiène hospitalière et de protection de l'environnement, Hôpital militaire principal d'instruction de Tunis (HMPIT), 1008 Montfleury, Tunis, Tunisie; 2 Service des maladies infectieuses, HMPIT; 3 Service de pneumo-phtisiologie, HMPIT; 4 Service d'anesthésie-réanimation, HMPIT; 5 Service d'accueil des urgences, HMPIT; 6 Service de gynécologie obstétrique, HMPIT; 7 Laboratoire de virologie, HMPIT; 8 Faculté de médecine de Tunis, Université Tunis El Manar, Tunisie

**Keywords:** SARS-CoV-2, COVID-19, Vagues épidémiques, Comorbidités, Tunisie, Maghreb, Afrique du Nord, SARS-CoV-2, COVID-19, Epidemic waves, Comorbidities, Tunisia, Maghreb, Northern Africa

## Abstract

**Introduction:**

Le nouveau coronavirus (SARS-CoV-2) a déclenché une pandémie mondiale avec des conséquences sanitaires qui n’étaient pas similaires lors des vagues successives qui ont touché plusieurs pays. Le but de notre étude était de comparer les caractéristiques socio-démographiques, cliniques et évolutives des patients atteints de la COVID-19 hospitalisés au cours des 2^e^ et 3^e^ vagues survenues en Tunisie.

**Patients et méthodes:**

Étude prospective observationnelle incluant 1 527 patients atteints de la COVID-19 et hospitalisés à l'Hôpital militaire de Tunis, s’étalant sur 11 mois, répartie en deux périodes : de juillet 2020 jusqu'en décembre 2020 appelée la deuxième vague (V2) et de janvier 2021 jusqu'en mai 2021 appelée la troisième vague (V3).

**Résultats:**

636 patients ont été hospitalisés au cours de V2 contre 891 au cours de V3. L’âge moyen était de 63,5 ± 15,3 ans au cours de V2 contre 65,8 ± 17,8 ans au cours de V3 (P = non significatif [NS]). Le sexe-ratio (H/F) était de 1,59 pour V2 et de 1,42 pour V3 (P = NS). La forme clinique sévère représentait 49% des cas pendant V2 contre 34,8% pendant V3 (P < 10^-3^). La forme critique représentait 18,6% des cas au cours de V2 contre 16,8% au cours de V3 (P = NS). Le taux de létalité était de 24,5% au cours de V2 et de 20,7% au cours de V3 (P = NS). L’âge médian des patients décédés était de 70,2 ans [42-88 ans] au cours de V2 et de 70,4 ans [22-96 ans] au cours de V3. Le sexe-ratio (H/F) des patients décédés était de 3,21 pour V2 et de 1,5 pour V3 (P = 0,001). Le taux de létalité était plus élevé dans le service de réanimation (65,4% pour V2 *versus* 69,7% pour V3; P = NS). Les causes de décès étaient dominées par le SDRA (syndrome de détresse respiratoire aiguë) pour les deux périodes (55,1% pour V2 *versus* 70,8% pour V3; P = 0,002), suivi de l’état de choc septique (12,8% pour V2 *versus* 10,8% pour V3; P = NS) et de la défaillance multiviscérale (9,6% pour V2 *versus* 7,0% pour V3; P = NS).

**Conclusion:**

Nous avons observé une diminution des formes cliniques sévères et critiques au cours de V3, ainsi qu'une baisse du taux de létalité par rapport à V2 grâce à l'amélioration de la prise en charge ainsi qu’à la vaccination. En revanche, le pourcentage de SDRA était significativement plus élevé au cours de V3 probablement en rapport avec le début de circulation dans notre pays de la souche Delta à l'origine de tableaux cliniques plus sévères.

## Abstract (en)

**Introduction:**

Since December 2019, a novel coronavirus (SARS-CoV-2) has triggered a global pandemic with a heavy medical and societal-economic toll. The health consequences were not similar during the successive waves that affected several countries. The aim of our study was to compare the sociodemographic, clinical and evolutionary features of COVID-19 patients hospitalized at the Military Hospital of Tunis (HMPIT) during the 2^nd^ and 3^rd^ waves that affected the country.

**Patients and methods:**

Observational prospective study involving 1,527 COVID-19 patients hospitalized at HMPIT over 11 months, divided into two periods: from July 2020 to December 2020 called the second wave (V2) and from January 2021 to May 2021 called the third wave (V3). We compared the epidemiological data, the clinical form and the evolution of the patients for each period.

**Results:**

The number of hospitalized patients was 636 during V2 compared to 891 during V3. Average age was 63.5 ± 15.3 years during V2 *versus* 65.8 ± 17.8 years during V3 (P = not significant [NS]). The percentage of young adults [18-40 years] was 6.5% during V2 compared to 6.7% during V3 (P = NS). The gender ratio (M/F) was 1.59 for V2 and 1.42 for V3 (P = NS). Comorbidities were present in 65% of V2 patients and 66.3% of V3 patients (P = NS), with hypertension being the most prevalent one in both groups (47.2% for V2 *versus* 44.9% for V3; P = NS), followed by overweight, dyslipidemia and diabetes (33% for V2 *versus* 39.3% for V3; P = 0.012). The median duration between symptoms onset and hospitalization was 7 days [5-10] during V2 *versus* 8.5 days during V3 [5-12] (P = 0.0004). The severe clinical form was present in 49% of patients admitted during V2 compared to 34.8% during V3 (P < 10^-3^). The critical form represented 18.6% of cases during V2 against 16.8% during V3 (P = NS). The average hospital length of stay in COVID units (outside of intensive care unit) was 8.4 ± 5.4 days during V2 and 9.8 ± 5.7 days during V3. The average length of stay was significantly longer for the intensive care unit (11.3 ± 3.4 days for V2 *versus* 13.8 ± 3.9 days for V3; P = 0.01). The case fatality rate was 24.5% during V2 and 20.7% during V3 (P = NS). Median age of death was 70.2 years [42-88] during V2 and 70.4 years [22-96] during V3 with 2 patients less than 40 years of age (1%) for the latter period. The gender ratio (M/F) of deceased patients was 3.21 for V2 and 1.5 for V3 (P = 0.001). The case fatality rate was higher in the intensive care unit (65.4% for V2 *versus* 69.7% for V3; P = NS). Causes of death were dominated by ARDS (acute respiratory distress syndrome) for both periods (55.1% for V2 *versus* 70.8% for V3; P = 0.002), followed by septic shock (12.8% for V2 *versus* 10.8% for V3; P = NS) and multi-organ failure (9.6% for V2 *versus* 7.0% for V3; P = NS).

**Conclusion:**

This study revealed a decrease in severe and critical clinical forms during the 3rd wave, as well as a decrease in the case fatality rate compared to the previous wave, due to improved management and vaccination. On the other hand, the percentage of ARDS was significantly higher during this wave probably related to the beginning of circulation in our country of the Delta variant causing more severe clinical cases.

## Introduction

Le coronavirus de type 2 responsable du Syndrome respiratoire aigu sévère (SARSCoV-2), initialement apparu à Wuhan en Chine, a été identifié comme l'agent causal de la maladie à coronavirus 2019 (COVID-19). Sa propagation pandémique à l’échelle mondiale depuis décembre 2019 a engendré un lourd bilan médical et socioéconomique [27, 28]. Ce virus possède comme particularité une transmission interhumaine très importante, surtout en cas de foule ou de rassemblement. Il touche toutes les tranches d’âge, avec une vulnérabilité particulière pour les sujets âgés et ceux ayant des comorbidités préexistantes [17]. Les conséquences sanitaires n’étaient pas similaires lors des vagues successives qui ont touché un certain nombre de pays [25]. Cette différence s'explique en partie par le degré et l'application des mesures préventives mises en oeuvre, le taux de couverture vaccinale contre la COVID, la virulence et le degré de contagiosité du SARS-CoV-2 tributaires en grande partie des mutations du virus émergent initial.

Le but de notre étude était de comparer les caractéristiques socio-démographiques, cliniques et évolutives des patients atteints de la COVID-19 hospitalisés à l'Hôpital militaire principal d'instruction de Tunis (HMPIT) au cours des 2^e^ et 3^e^ vagues qui ont touché le pays, et de déterminer les principaux facteurs de risque de morbidité et de mortalité de ces patients.

## Patients et Méthodes

Nous avons mené une étude prospective observationnelle incluant 1 527 patients atteints de la COVID-19 et hospitalisés à l'HMPIT, s’étalant sur 11 mois, répartie en deux périodes : de juillet 2020 jusqu'en décembre 2020 appelée la deuxième vague (V2) et de janvier 2021 jusqu'en mai 2021 appelée la troisième vague (V3). Cette dernière vague correspondait à la période où la vaccination avait débuté en Tunisie (mars 2021) et où la souche Alpha était dominante [2], remplaçant la souche d'origine de Wuhan introduite en Tunisie en mars 2020. L'HMPIT constitue le principal et le plus grand hôpital militaire pluridisciplinaire du pays, couvrant l'ensemble des 24 gouvernorats du pays pour les militaires et leurs familles. Cependant, 20% de sa capacité de prise en charge est réservée à des civils tunisiens.

Pour chaque période ont été recueillis à partir de dossiers médicaux : les données socio-démographiques et épidémiologiques (âge, sexe, lieu de résidence, catégorie de patient, date de début de la COVID, date de diagnostic, date d'hospitalisation, date de sortie ou de décès) et médicales (antécédents médicaux, forme clinique et évolution des patients). Ces données ont été analysées globalement sur l'ensemble des 1 527 patients puis comparées selon la vague épidémique (V2 *versus* V3).

Le diagnostic de la COVID-19 était retenu sur la positivité de la RT-PCR SARS-CoV-2 sur un prélèvement nasopharyngé ou en cas de négativité de celle-ci, sur un faisceau d'arguments épidémiologiques associés à des lésions pulmonaires évocatrices au scanner.

Les formes cliniques étaient classées en :

- Forme modérée : définie par la présence de signes d'infection respiratoire basse (fièvre, toux, discrète dyspnée) sans signes de sévérité;
- Forme sévère : définie par la présence d'une symptomatologie d'infection respiratoire basse avec un ou plusieurs signes de sévérité (dyspnée, fréquence respiratoire > 30 cycles/mn, taux de saturation en oxygène (SpO2) ≤ 92% à l'air ambiant, infiltrats pulmonaires > 50% au scanner thoracique);
- Forme critique : définie par la présence d'un syndrome de détresse respiratoire aiguë (SDRA) associé ou non à une défaillance d'autres organes.

Les unités COVID à l'HMPIT étaient organisées comme suit :

- L'unité COVID du service d'accueil des urgences (prise en charge des patients COVID avant leur transfert aux autres unités);
- L'unité COVID-réanimation;
- Les services transformés en unités COVID aux étages :

- services de médecine (maladies infectieuses, pneumologie, psychiatrie, dermatologie);
- services chirurgicaux (ORL, chirurgie vasculaire).

Les patients étaient accueillis et examinés aux urgences, puis étaient dispatchés en fonction de la gravité du tableau clinique : les patients présentant une forme clinique légère n’étaient pas hospitalisés, un traitement symptomatique leur a été prescrit. Les formes cliniques modérées étaient prises en charge dans les services transformés en unités COVID aux étages, et les formes critiques étaient transférées directement à l'unité COVID-réanimation.

En plus du traitement symptomatique, un traitement anticoagulant était prescrit pour tous les patients hospitalisés pendant toute la durée de l'hospitalisation, la dose et la durée étaient adaptées en fonction de la sévérité de la maladie, des facteurs de risque thromboemboliques et de la fonction rénale.

Les formes modérées sans facteurs de risque thromboemboliques ont bénéficié de la dose préventive, les formes modérées avec facteurs de risque et les formes sévères ou critiques avec ou sans facteurs de risque ont bénéficié de la dose iso-coagulante. La dose curative a été prescrite en cas de D-dimères > 3 000 UI ou score SIS (Sepsis-Induced Coagulopathy) ≥ 4 ou antécédents de thrombose.

L'oxygénothérapie était indiquée en cas de désaturation avec une diminution de la pression partielle en oxygène à moins de 92% à l'air ambiant.

La corticothérapie était indiquée dans les formes cliniques sévères et critiques nécessitant une oxygénothérapie. Un traitement antibiotique n’était prescrit qu'en présence d'arguments clinico-biologiques et/ou radiologiques de surinfection bactérienne.

Les différentes données ont été saisies et analysées au moyen du programme EXCEL et SPSS version 19.2.

Les comparaisons de 2 moyennes sur séries indépendantes ont été effectuées au moyen du test t de Student pour séries indépendantes. Les comparaisons de pourcentages sur séries indépendantes ont été effectuées par le test du chi-deux de Pearson, et en cas de non-validité de ce test par le test exact bilatéral de Fisher. Les liaisons entre 2 variables quantitatives ont été étudiées par le coefficient de corrélation de Pearson, et en cas de non-validité par le coefficient de corrélation des rangs de Spearman. Le seuil de signification a été fixé à 0,05.

## Résultats

Pendant la 2^e^ vague (V2), 636 patients COVID ont été hospitalisés, dont 4 femmes enceintes (0,6%), 4 enfants (0,6%) et un nouveau-né (0,1%). Pendant la 3^e^ vague (V3), 891 patients ont été hospitalisés dont 16 femmes enceintes (1,7%), 6 enfants (0,6%) et un nouveau-né (0,1%).

La répartition des patients selon les unites de prise en charge est représentée dans le Tableau I.

**Tableau I:** Répartition des 1 527 patients hospitalisés pour COVID-19 à l'Hôpital militaire principal d'instruction de Tunis (HMPIT) selon les unités et la vague épidémique Distribution of the 1 527 patients hospitalized for COVID-19 at the Military Hospital of Tunis according to COVID unit and epidemic wave

	2^e^ vague juillet-décembre 2020 N=636 (%)	3^e^ vague janviermai 2021 N=891 (%)	P
**Unité Covid-urgences**	188 (29,6%)	137 (15,4%)	<10^-3^
**Unité Covid-réanimation**	118 (18,6%)	15 (16,9%)	NS
**Services de Médecine**	259 (40,7%)	461 (51,7%)	<10^-3^
**Services Chirurgicaux**	71 (11,2%)	142 (16,0%)	0,0079

NS : non significatif

### Caractéristiques socio-démographiques

L’âge médian de l'ensemble des patients hospitalisés au cours des deux vagues était de 64,9 ans [1 mois-96 ans].

**Figure 1:**
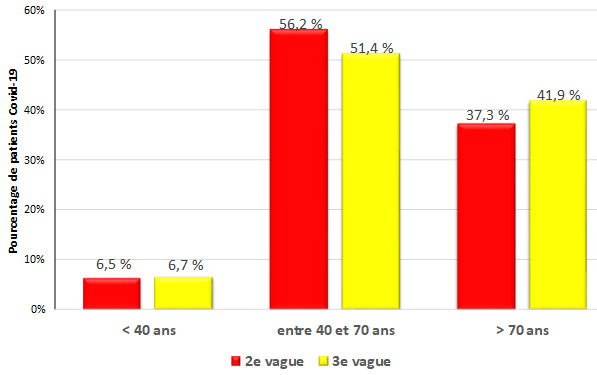
Répartition des 1 527 patients hospitalisés pour COVID-19 de l'HMPIT selon les tranches d’âge et les vagues épidémiques Distribution of the 1 527 patients hospitalized for COVID-19 of the HMPIT according to age group and epidemic wave

L’âge moyen des patients était de 63,5 ans ± 15,3 au cours de V2 contre 65,8 ans ± 17,8 pendant V3 (P = non significatif [NS]). Cent deux patients (6,67%) étaient des adultes jeunes [18-39 ans]. Ces derniers représentaient 6,5% et 6,7% de l'ensemble des patients au cours de V2 et V3 respectivement (P = NS). La tranche d’âge la plus représentée au sein de notre série était celle de 40 à 70 ans avec un pourcentage de 56,2% au cours de V2 et de 51,4% au cours de V3 (Fig. 1). Le sexe-ratio (H/F) était égal à 1,59 pour V2 et 1,42 pour V3 sans qu'il y ait de différence statistiquement significative (Tableau II).

**Tableau II:** Répartition des 1 527 patients hospitalisés pour COVID-19 à l'Hôpital militaire principal d'instruction de Tunis (HMPIT) selon le genre et la vague épidémique Distribution of the 1 527 patients hospitalized for COVID-19 at the Military Hospital of Tunis according to gender and epidemic wave

	2^e^ vague juillet-décembre 2020 N=636 (%)	3^e^ vague janviermai 2021 N=891 (%)	P
**Hommes**	391 (61,5%)	523 (58,7%)	
**Femmes**	245 (38,5%)	368 (41,3%)	
**Sexe-ratio**	1,59	1,42	NS

NS : non significatif

**Figure 2:**
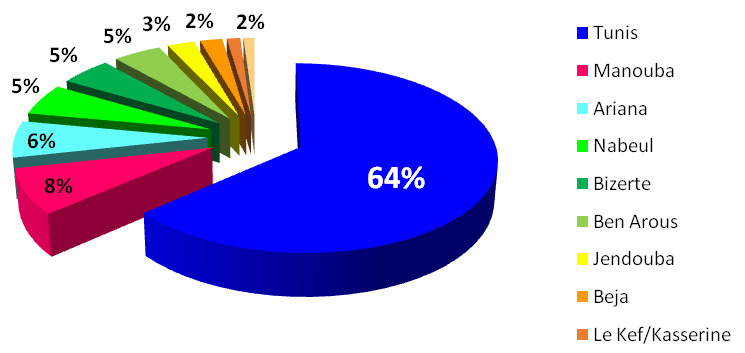
Répartition des 1 527 patients hospitalisés pour COVID-19 de l'HMPIT selon l'origine géographique (par gouvernorat) Distribution of the 1 527 patients hospitalized for COVID-19 of the HMPIT according to geographical origin (by governorate)

La majorité des patients (78%) provenaient du grand Tunis, regroupant la capitale et les gouvernorats frontaliers (Manouba, Ariana, Ben Arous) (Fig. 2). Le nombre de patients non militaires admis et ayant une couverture sociale était significativement plus élevé au cours de V3 que V2 (35,3% *versus* 26,2%; P < 10^-3^) (Tableau III).

**Tableau III:** Répartition des 1 527 patients hospitalisés pour COVID-19 à l'Hôpital militaire principal d'instruction de Tunis (HMPIT) selon la catégorie de patient et la vague épidémique Distribution of the 1 527 patients hospitalized for COVID-19 at the Military Hospital of Tunis according to category of patient and epidemic wave

	2^e^ vague juillet-décembre 2020 N = 636 (%)	3^e^ vague janvier-mai 2021 N = 891 (%)	P
**Ayant droit**	444 (69,8%)	553 (62,1%)	0,0017
CNAM*	167 (26,3%)	315 (35,4%)	0,00016
**Civil payant**	25 (3,9%)	23 (2,6%)	NS

NS : non significatif

* Caisse nationale d'assurance maladie

### Caractéristiques médicales

Soixante-cinq pour cent des patients de la V2 avaient au moins un antécédent médical contre 66,3% pour la V3 (P = NS). L'hypertension artérielle était l'antécédent le plus fréquent dans les deux périodes (47,2% pour V2 *versus* 44,9% pour V3; P = NS), suivie par le surpoids, la dyslipidémie et le diabète (33,0% pour V2 contre 39,3% pour V3; P = 0,012) (Tableau IV).

**Tableau IV:** Répartition des 1 527 patients hospitalisés pour COVID-19 à l'Hôpital militaire principal d'instruction de Tunis (HMPIT) en fonction des antécédents médicaux et de la vague épidémique Distribution of the 1 527 patients hospitalized for COVID-19 at the Military Hospital of Tunis according to medical history and epidemic wave

	**2^e^ vague juillet-décembre 2020 N = 636 (%)**	**3^e^ vague janvier-mai 2021 N = 891 (%)**	**P**
**Sans antécédents médicaux avant la COVID-19**	223 (35,0%)	300 (33,7%)	NS
**Avec un ou plusieurs antécédents médicaux avant la COVID-19**	413 (65,0%)	591 (66,3%)	NS
**HTA**	300 (47,2%)	400 (44,9%)	NS
**Surpoids**	254 (40,0%)	395 (44,3%)	NS
**Dyslipidémie**	220 (34,6%)	300 (33,7%)	NS
**Diabète**	210 (33,0%)	350 (39,3%)	0,012
**Coronaropathie**	38 (6,0%)	65 (7,3%)	NS
**Insuffisance cardiaque**	25 (3,9%)	40 (4,5%)	NS
**AVC/AIT(a)**	13 (2,1%)	15 (1,7%)	NS
**BPCO(b)**	28 (4,4%)	45 (5,0%)	NS
**Insuffisance rénale**	20 (3,1%)	35 (3,9%)	NS

NS : non significatif

(a) Accident ischémique transitoire

(b) Bronchopneumopathie chronique obstructive

**Figure 3:**
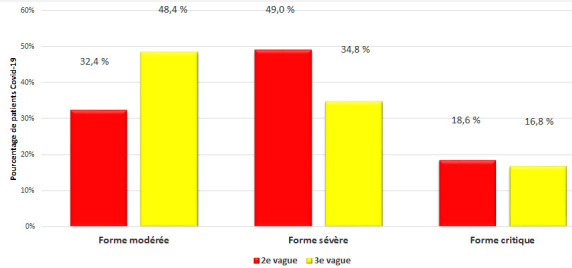
Répartition des 1 527 patients hospitalisés pour COVID-19 de l'HMPIT selon la sévérité du tableau clinique et les périodes épidémiques Distribution of the 1 527 patients hospitalized for COVID-19 of the HMPIT according to clinical form of severity and epidemic period

Le pourcentage des patients ayant une forme clinique modérée était significativement plus élevé pour V3 que pour V2 (48,4% contre 32,4%; P < 10^-3^). La forme clinique sévère était présente à l'admission chez 49,0% des patients admis pendant V2 contre 34,8% pendant V3 (P < 10^-3^). Le pourcentage des patients présentant une forme critique à l'admission était de 18,6% et de 16,8% respectivement au cours de V2 et V3, sans différence significative (P = NS) (Fig. 3).

Le délai médian entre le début des signes cliniques et le recours au système de santé était de 7 jours [5-10] pour V2 contre 8,5 jours pour V3 [5-12] (P = 0,0004). La durée moyenne d'hospitalisation dans les unités COVID (en dehors de la réanimation) était de 8,4 ± 5,4 jours pendant V2 et de 9,8 ± 5,7 jours pendant V3 (P = 0,03). La durée moyenne du séjour était significativement plus longue pour le service de réanimation (11,3 ± 3,4 jours pour V2 contre 13,8 ± 3,9 jours pour V3) (P = 0,01) (Tableau V).

**Tableau V:** Durée moyenne d'hospitalisation des patients COVID de l'HMPIT en jours selon les services de prise en charge et les périodes épidémiques Average length of hospitalization of HMPIT COVID patients in days according to management service and epidemic period

Services	2^e^ vague	3^e^ vague	P
**Réanimation**	11,3 jours	13,8 jours	0,01
**Hors réanimation**	8,4 jours	9,8 jours	0,03
**Médecine**	8,9 jours	10,3 jours	NS
**Chirurgie**	8,0 jours	9,3 jours	NS
**Unité COVID des urgences**	3,0 jours	1,9 jours	0,02

NS : non significatif

Les complications les plus fréquentes étaient la pneumopathie sévère et le SDRA (43,0% au cours de V2 contre 40,0% au cours de V3; P = NS), suivis du sepsis selon l'ancienne définition (11,0% au cours de V2 contre 10,0% au cours de V3; P = NS) puis des complications thromboemboliques (8,0% au cours de V2 contre 7,0% au cours de V3; P = NS).

### Caractéristiques des patients décédés

Le taux de létalité globale était égal à 22,3% (341/1527). Il était égal à 24,5% (156/636) au cours de V2 et à 20,7% (185/891) au cours de V3 sans différence statistiquement significative (P = NS).

L’âge médian des patients décédés était de 70,2 ans [42-88] au cours de V2 et de 70,4 ans [22-96] au cours de V3 avec 2 patients âgés de moins de 40 ans (1%) pour cette dernière période.

**Figure 4:**
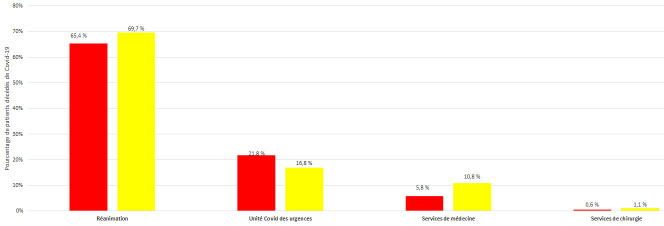
Répartition des 341 patients décédés de COVID-19 à l'HMPIT selon les services et les périodes épidémiques Distribution of the 341 patients who died from COVID-19 at the HMPIT according to service and epidemic period

Le sexe-ratio (H/F) des patients décédés était de 3,21 (119 H/37 F) pour V2 et de 1,5 (111 H/74 F) pour V3 (P = 0,001). Le taux de létalité pour les hommes était significativement plus élevé au cours de V2 (30,4% (119/391) contre 21,2% (111/523) au cours de V3; P = 0,001). Pour les femmes, ce taux était de 15,1% (37/245) au cours de V2 et de 20,1% (74/368) au cours de V3 (P = NS). Le taux de létalité était plus élevé dans le service de réanimation (65,4% pour V2 *versus* 69,7% pour V3; P = NS), suivi de l'unité COVID des urgences (21,8% pour V2 *versus* 16,8% pour V3; P = NS), des unités COVID des services de médecine (5,8% pour V2 *versus* 10,8% pour V3 avec une différence statistiquement significative : P = 0,04). Les unités COVID des services chirurgicaux avaient le taux de létalité le plus faible (0,6% pour V2 contre 1,1% pour V3; P = NS) (Fig. 4).

Les causes de décès étaient dominées par le SDRA pour les deux périodes (55,1% pour V2 *versus* 70,8% pour V3; P = 0,002), suivi de l’état de choc septique (12,8% pour V2 *versus* 10,8% pour V3; P = NS) et de la défaillance multiviscérale (9,6% pour V2 *versus* 7,0% pour V3; P = NS) (Tableau VI).

**Tableau VI:** Principales causes de décès des 341 patients hospitalisés pour COVID-19 de l'HMPIT selon les vagues épidémiques Major causes of death for the 341 patients hospitalized for COVID-19 at HMPIT according to epidemic wave

	2^e^ vague N=156 (%)	3^e^ vague N = 185 (%)	P
**Pneumopathie sévère/SDRA(a)**	86 (55,1%)	131 (70,8%)	0,002
**État de choc septique**	20 (12,8%)	20 (10,8%)	NS
**Défaillance multiviscérale**	15 (9,6%)	13 (7,0%)	NS
**Hypoxie**	15 (9,6%)	10 (5,4%)	NS
**Complications thromboemboliques**	6 (3,9%)	3 (1,6%)	NS
**État de choc cardiogénique**	4 (2,6%)	2 (1,1%)	NS
**Insuffisance rénale aiguë**	3 (1,9%)	1 (0,5%)	NS
**État de choc hémorragique**	2 (1,3%)	3 (1,6%)	NS
**AVC hémorragique**	1 (0,6%)	2 (1,1%)	NS
**Œdème aigu du poumon**	2 (1,3%)	0 (0,0%)	NS
**Insuffisance hépatocellulaire/pancréatite aiguë**	2 (1,3%)	0 (0,0%)	NS

NS : non significatif

(a) Syndrome de détresse respiratoire aiguë

(b) Bronchopneumopathie chronique obstructive

## Discussion

Jusqu'en mai 2020, plus de 3,3 millions de cas confirmés de COVID-19 et plus de 230 000 décès attribuables à la maladie ont été signalés dans le monde [21]. À cette date, la Tunisie était relativement épargnée au cours de la première vague (45 décès en 3 mois) du fait qu'elle n'a pas hésité à instaurer un confinement national d'une durée de 3 mois dès l'apparition des premiers cas en mars 2020. Cette mesure n'a pu éviter les conséquences des vagues ultérieures surtout celles du début d'année 2021, correspondant à la 3^e^ vague et causant en moyenne 70 morts par jour avec un premier pic de 103 décès le 22 janvier, puis un deuxième pic de 119 décès le 28 avril. Le pic maximal a été observé lors de la 4e vague qui avait débuté en juillet 2021, avec un total de 381 décès le 1^er^ août [6]. Cette dégradation de la situation sanitaire s'expliquerait par :

- le relâchement des mesures barrières;
- l'allégement des mesures de contrôle au niveau des frontières;
- les rassemblements lors des mouvements sociaux;
- un manque de ressources en milieu hospitalier;
- le retard de la vaccination anti-COVID, la campagne vaccinale n'ayant commence que le 13 mars 2021 alors que celle des pays voisins a été lancée dès janvier;
- l'introduction, en avril-mai 2021, de la souche Delta, plus contagieuse et plus virulente que les précédentes.

Une connaissance approfondie des signes cliniques, biologiques et radiologiques associés à la maladie est essentielle pour améliorer la prise en charge. Un nombre considérable d’études et de recherches ont été de ce fait réalisées, dont une métaanalyse de 148 études menées en Chine (près de 90% des études incluses dans la méta-analyse), en Europe et aux États-Unis, publiée en octobre 2020, portant sur plus de 12 000 patients et fournissant un aperçu détaillé sur les particularités épidémiologiques, cliniques, thérapeutiques et évolutives chez les patients adultes, les femmes enceintes et les enfants atteints de la COVID-19 [17]. Selon cette méta-analyse, l’âge médian des patients hospitalisés était de 47 ans [35-65 ans] [17]. Dans notre série, l’âge médian des patients hospitalisés était significativement plus élevé (64,9 ans), ce qui pourrait s'expliquer par l'inclusion des patients âgés de plus de 65 ans et la rareté de la population pédiatrique dans notre étude. L’âge moyen des patients était de 63,5 ± 15,3 ans au cours de V2 contre 65,8 ± 17,8 ans pendant V3. La majorité des patients étaient âgés entre 40 et 70 ans au cours des deux vagues. Nos résultats rejoignent ceux retrouvés dans une étude réalisée en Algérie entre mai 2020 et août 2021 comparant le profil clinico-biologique et évolutif entre les 2^e^ et 3^e^ vagues de la COVID-19 et où la moyenne d’âge était respectivement de 60 ans et 54 ans [26].

Dans une étude réalisée au Maroc, l’âge médian des patients hospitalisés pendant V2 entre juillet 2020 et janvier 2021 était de 51 ans [45-59] et 43% d'entre eux étaient âgés entre 43 et 54 ans [8].

En France, 47% des patients hospitalisés pendant V2 étaient âgés de plus de 75 ans et 21% avaient entre 30 et 59 ans [7].

Le sexe-ratio (H/F) dans les 2 périodes comparées rejoint l'ensemble des études de la méta-analyse qui avaient noté une proportion significativement plus élevée d'hommes atteints que de femmes (P = 0,008) [17].

Dans notre étude, les comorbidités étaient présentes chez 65% des patients pendant V2 et chez 66,3% pendant V3. Nos résultats étaient significativement plus élevés que ceux de la méta-analyse où la fréquence globale des patients avec au moins une comorbidité était deux fois moins élevée, égale à 31%. L'hypertension artérielle était l'antécédent le plus fréquent (20,9%), puis l'insuffisance cardiaque (10,5%), le diabète (10,4%) et l'insuffisance coronarienne (8,5%). Ceci s'explique en partie par un âge médian de l'ensemble des patients moins élevé que celui de notre étude [17]. Les comorbidités préexistantes sont liées à la fois à la sévérité et à la mortalité de la maladie. La fréquence plus élevée des comorbidités de notre étude liée à l’âge explique une fréquence des formes cliniques critiques (18,6% et 16,8% selon la vague épidémique) et une létalité (22,3%) plus élevées que celles de la méta-analyse (respectivement 6,8% pour la fréquence des formes cliniques critiques et 8,0% pour la létalité) [17].

Le nombre de patients hospitalisés au cours de la 3^e^ vague était plus élevé par rapport à la vague précédente (891 en 5 mois soit une moyenne de 178 hospitalisations/mois, contre 636 en 6 mois soit une moyenne de 106 hospitalisations/mois). Ceci pourrait s'expliquer en partie par le début de la circulation de la souche Delta.

Le pourcentage de patients hospitalisés sous une forme clinique sévère était significativement plus important pendant la V2 que la V3. En effet, au cours de la V3, le nombre de formes sévères avait significativement diminué, laissant place aux formes modérées de la maladie. Nos résultats rejoignent ceux retrouvés dans l’étude de Rousseau *et al.* réalisée au Centre hospitalier intercommunal de Créteil incluant 1 076 patients hospitalisés, où le pourcentage de formes sévères était passé de 31% pendant la 2^e^ vague à 18% pendant la 3^e^ vague [25]. De même pour le pourcentage de patients admis avec une forme critique au service de réanimation, ce taux s’étant sensiblement amélioré au cours de la V3 par rapport à la V2, sans différence significative (P = NS). Ceci pourrait s'expliquer par l'amélioration de la prise en charge des patients par les médecins de première ligne. La 3^e^ vague correspond aussi à la période de la première phase du plan national de vaccination anti-COVID qui avait débuté en mars 2021 et qui avait ciblé le personnel de santé et les personnes âgées. Cet effet de la vaccination était également visible au Maroc, qui avait connu une troisième vague certes plus rapide mais moins létale et avec moins de formes sévères [11]. En effet, malgré la hausse exponentielle des nouveaux cas au Maroc, le taux de létalité était plus faible comparé à celui observé en 2020. En novembre 2020 au pic de la 2^e^ vague, le nombre des décès était égal à 92 en 24 heures, contre 32 au cours de la 3^e^ vague. La campagne de vaccination était alors bien avancée dans le pays [11].

Nos résultats ont objectivé une diminution du taux de létalité au cours de la V3 par rapport à la V2. Ceci pourrait s'expliquer par une meilleure connaissance de la physiopathologie de la maladie, une amélioration de la prise en charge secondaire aux avancées scientifiques et l’échange d'informations entre les différentes équipes dans le monde. De plus, la population ayant survécu aux deux premières vagues est supposée avoir un meilleur statut sanitaire et immunitaire.

L’âge moyen des patients décédés dans notre étude était de 70,2 ans. Deux adultes jeunes de moins de 40 ans (1%) étaient décédés pendant V3. En effet, les adultes jeunes n’étaient pas encore vaccinés pendant cette période en Tunisie. La proportion d'hommes décédés était significativement plus élevée que celle des femmes.

Nos résultats rejoignent ceux de la littérature, où la majorité des études avait conclu que l’âge avancé [23, 30] et le sexe masculin [15] étaient des facteurs de risque prédictifs de sévérité et de mortalité de la maladie. Ceci s'expliquait par le processus de vieillissement et le déclin du système immunitaire, surtout la fonction des lymphocytes T et B, et la production excessive de cytokines de type 2 [10, 19]. Ce vieillissement immunitaire est en effet soupçonné d'entraîner une déficience dans le contrôle de la réplication virale et des réponses pro-inflammatoires plus prolongées, de mauvais pronostic [22].

Plusieurs études ont également constaté l'existence d'une différence entre les 2 sexes en matière de mortalité et de vulnérabilité à la maladie [14, 29]. Les hommes étaient effectivement plus fréquemment touchés par la maladie et leur mortalité à l'hôpital était significativement plus élevée que chez les femmes. Plusieurs hypothèses ont été proposées, notamment les différences dans le système immunitaire [5], le polymorphisme génétique [9], les facteurs liés au mode de vie, y compris le tabagisme [12, 18], les habitudes d'hygiène personnelle [12, 16], les comorbidités préexistantes [20, 24] et l'expression de l'enzyme de conversion de l'angiotensine 2 (ACE2) [1, 13]. Cette différence de vulnérabilité entre les sexes a également été observée pour le SARS-CoV-1 et le MERS [4], deux maladies à coronavirus précédemment émergentes.

Le service de réanimation avait le pourcentage de décès le plus élevé par rapport aux autres services, s'expliquant par la nature des patients admis majoritairement dans des situations critiques. Le recrutement d'emblée des patients les moins graves explique le taux de létalité plus faible dans les unités COVID des services chirurgicaux de notre hôpital. Nous avions noté une amélioration du taux de létalité enregistré dans l'unité COVID du service des urgences au cours de la V3 par rapport à la vague précédente. La pneumopathie sévère et le SDRA étaient les complications les plus fréquentes et les causes de décès prédominantes. Le pourcentage de SDRA était significativement plus élevé au cours de la V3 (70,8%) par rapport à la V2 (55,1%), ceci pouvant s'expliquer par le début de circulation de la souche Delta dans notre pays qui est à l'origine de formes cliniques rapidement progressives, ainsi que par le retard de consultation des patients observé dans notre étude entre V2 et V3. En effet, d'après le bilan du ministère de la Santé, pour la seule journée du 7 avril 2021, la Tunisie avait enregistré 1 833 nouvelles contaminations sur 7 364 tests effectués (soit un taux de positivité de 24,9%). À cette date il y avait 1 844 patients hospitalisés. Parmi eux, 117 ont été placés sous respirateurs artificiels (6,3%) et 388 ont été admis en soins intensifs (21%) [2]. Le ministère de la Santé avait alors signalé l'apparition du variant Delta de la COVID-19 avec 133 hospitalisations supplémentaires en 24 h aussi bien dans les structures de santé publiques que privées [2].

Selon une étude réalisée au CHU de Béni Messous à Alger [3], 3 064 patients avaient été hospitalisés pendant V2 et V3, 646 décès avaient été enregistrés soit un taux de létalité hospitalière globale de 21,1% comparable à celui de notre étude. La mortalité était significativement plus élevée chez les patients âgés de plus de 65 ans, diabétiques et ayant des antécédents de maladies respiratoires et cardiovasculaires (P < 0,001) [3].

## Conclusion

Au terme de notre étude, nous pouvons conclure que le sexe masculin, l’âge avancé et les comorbidités préexistantes étaient des facteurs de risque majeurs de survenue de complications et de mortalité des patients atteints de la COVID-19 hospitalisés.

Le taux élevé de létalité hospitalière de la COVID-19 (22,3%) dans notre etude pourrait s'expliquer au total par différents facteurs : la prise de décision de la cellule de crise de notre hôpital d'augmenter le nombre de lits de réanimation (35 lits) pour prendre en charge plus de formes critiques et participer ainsi à l'effort national de lutte contre la COVID-19, la fréquence élevée des comorbidités (65%) et le retard à la consultation.

La 3^e^ vague était particulièrement marquée par une diminution des formes cliniques sévères et critiques, ainsi qu'une baisse du taux de létalité par rapport à la vague précédente et ceci grâce à l'amélioration de la prise en charge et à la vaccination. En revanche, le pourcentage de SDRA était significativement plus élevé au cours de cette vague, probablement en rapport avec le début de circulation dans notre pays de la souche Delta à l'origine de tableaux cliniques plus sévères.

## Liens D'intérêt

Les auteurs ne déclarent aucun lien d'intérêt.

## Sources de Financement

Ce travail n'a fait l'objet d'aucun financement.

## Contribution des Auteurs

Rim RACHDI : conception de l’étude, recueil des données, élaboration des résultats statistiques, rédaction de l'article.

Sabrine ZRIBI et Oumaima AYED : recueil des données, élaboration des résultats statistiques.

Souha HANNACHI, Rim ABID, Zied MOATEMRI, Samira MHAMDI, Salsabil DABBOUSSI, Hédi GHARSALLAH, Walid SELLAMI, Walid SAMMOUD, Hakim MASSOUDI, Khaled LAMINE, Olfa DJEBBI, Rim HAMMAMI, Mohamed BEN MOUSSA, Ridha BELLAAJ, Riadh BATTIKH, Mohamed Radhouane RACHDI, Mustapha FERJANI : correction et validation finale du manuscrit.
